# Case Report: *Streptococcus dysgalactiae* ssp. *dysgalactiae* bloodstream infections in patients with breast cancer after radiotherapy and chemotherapy

**DOI:** 10.3389/fmed.2025.1572998

**Published:** 2025-05-30

**Authors:** Chongmei Tian, Su Dong, Zhixin You, Yaping Zhao, Hongfeng Xu, Jingbai Chen, Yuejuan Fang

**Affiliations:** ^1^Department of Pharmacy, Shaoxing Hospital of Traditional Chinese Medicine Affiliated to Zhejiang Chinese Medical University, Shaoxing, Zhejiang, China; ^2^State Key Laboratory of Neurology and Oncology Drug Development, Nanjing, Jiangsu, China; ^3^Department of Clinical Laboratory, Shaoxing Hospital of Traditional Chinese Medicine Affiliated to Zhejiang Chinese Medical University, Shaoxing, Zhejiang, China; ^4^Department of Respiratory Medicine, Shaoxing Hospital of Traditional Chinese Medicine Affiliated to Zhejiang Chinese Medical University, Shaoxing, Zhejiang, China; ^5^Pharmacy Department of Chinese Medicine, Shaoxing Hospital of Traditional Chinese Medicine Affiliated to Zhejiang Chinese Medical University, Shaoxing, Zhejiang, China; ^6^Department of Pharmacy, Quzhou Maternal and Child Health Care Hospital, Quzhou, China

**Keywords:** *Streptococcus dysgalactiae* ssp. *dysgalactiae*, bloodstream infections, breast cancer, *Streptococcus dysgalactiae*, whole genome sequencing analysis

## Abstract

Bloodstream infections are life-threatening conditions in patients with breast cancer, especially among older individuals. Although the most common risk factor in these patients with tumors is the use of severe neutropenia secondary to myelosuppressive chemotherapy and radiotherapy, other factors are possibly associated with the invasive pathogenicity of microorganisms, including *Streptococcus dysgalactiae*. *Streptococcus dysgalactiae* ssp. *dysgalactiae (SDSD)* has been considered both an environmental pathogen and a contagious pathogen. However, there have been few reports of bloodstream infections with SDSD in patients with breast cancer after radiotherapy and chemotherapy. In this study, we report an interesting case of bloodstream infection caused by SDSD in an older patient with breast cancer after radiotherapy and chemotherapy. A 60-year-old Chinese woman had a history of breast cancer for 2 years. She developed chills and fever after puncturing blood blisters in the mouth, accompanied by fatigue and poor appetite. After 6 days of antimicrobial therapy, the patient showed gradual recovery. Bloodstream infections with SDSD in patients with breast cancer are rare. Therefore, accurate diagnosis and timely treatment can be lifesaving.

## Introduction

*Streptococcus dysgalactiae* is a Gram-positive bacterium that is categorized into *Streptococcus dysgalactiae* ssp. *dysgalactiae* (SDSD) and *Streptococcus dysgalactiae* ssp. *equisimilis* (SDSE) (1). Invasive infections induced by *Streptococcus pyogenes* from group A streptococcus, *Streptococcus agalactiae* from group B streptococcus, and β-hemolytic streptococci from Lancefield groups A and B are reported to be increasing rapidly worldwide (2). Other streptococci include groups C, G, F, and L (3). Group G streptococci are remarkable because they have been shown to cause beta-hemolytic streptococcal bacteremia (4). According to previous studies, this group includes *S. dysgalactiae, Streptococcus anginosus*, and *Streptococcus canis* (5). Malignancy, diabetes mellitus, cardiovascular disease, cirrhosis, and bone and joint diseases are the most common underlying conditions in patients with group G beta-hemolytic streptococcal bacteremia (6, 7).

SDSD is generally described as α-hemolytic group C streptococci (8) that has been considered both an environmental pathogen and a contagious pathogen (9). It is a vital pathogenic microorganism of the bovine udder with the unique ability to induce severe clinical mastitis (10, 11). Mastitis occurs when a bacterial pathogen gains access to the mammary glands through the teat canal (12). SDSD belongs to a significant pathogenic group that belongs to the most common and costly cause of bovine mastitis (13). Despite this relatively high prevalence, little is known about the host factors and bacteria that contribute to the persistence and establishment of bloodstream infections caused by SDSD, and the natural reservoir of the bacteria. Therefore, the epidemiological characteristics and reservoirs of this bacteremia-causing bacterium require further investigation.

In this study, we discuss an interesting case of SDSD bloodstream infection in a patient with breast cancer after radiotherapy and chemotherapy. This finding has not been observed or reported in older patients with SDSD bloodstream infections with breast cancer after radiotherapy and chemotherapy, resulting in difficult therapeutic management in the present case. Clinicians should be aware of the clinical dilemmas and unusual presentations that arise during the treatment of such rare bloodstream infections, particularly in resource-limited settings.

## Case presentation

A 60-year-old Chinese woman was admitted to the Emergency Department of Shaoxing Hospital of Traditional Chinese Medicine affiliated with Zhejiang Chinese Medical University on 3 November 2024. One day prior to hospital admission, the patient developed intense chills (duration: 30–60 min) followed by intermittent high-grade fever peaking at 40.7°C (tympanic), with a diurnal variation of 1.5–2.0°C. These symptoms occurred 72 h after self-induced puncture of a 0.5 cm oral blood blister using a toothpick. Concurrently, she reported progressive fatigue, evidenced by a decline in Eastern Cooperative Oncology Group (ECOG) performance status from 1 (baseline) to 3 over 72 h, rendering her unable to perform basic activities of daily living. Poor appetite was exhibited as a 75% reduction in oral intake (estimated by 24-h dietary recall), followed by a 2.2-kg weight loss within 4 days.

**Figure 1:**
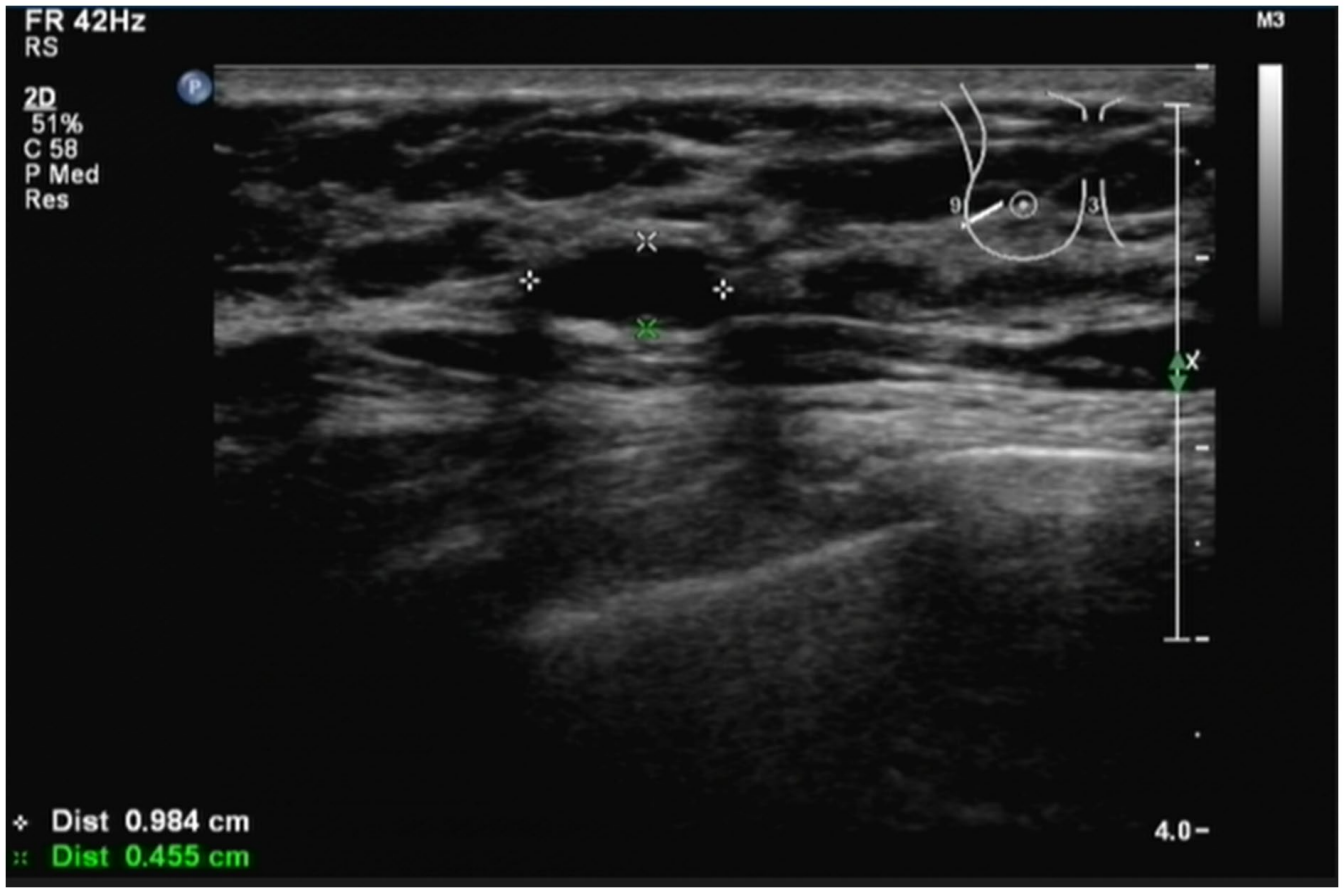
Patient was diagnosed with breast cancer by B-ultrasound. B-ultrasound examination showed that the size of the tumor in the left breast was 0.984 cm*0.455 cm. The patient had a history of breast cancer for 2 years.

The patient had a history of breast cancer for 2 years. A B-ultrasound examination showed that the tumor in the left breast measured 0.984 cm*0.455 cm (Figure 1) in December 2022. Therefore, the patient was diagnosed with breast cancer at the present hospital. The patient received eight cycles of chemotherapy at another teaching hospital in the province. She underwent left breast lymph node dissection in January 2023, and local radiotherapy was administered 25 times after surgery. Postoperative maintenance therapy included exemestane 25 mg daily and abemaciclib 100 mg two times daily. She had no history of coronary heart disease, blood disease, tuberculosis, hepatitis, kidney disease, mental health disorders, family history of breast/ovarian cancer, or hereditary syndromes. She also denied a history of depression, substance use, or tobacco/alcohol consumption. The patient had a 10-year history of type 2 diabetes mellitus, managed with acarbose 100 mg orally three times daily (TID). She self-reported good glycemic control, though no recent HbA1c data were available. In addition, she had a 10-year history of hypertension, treated with losartan potassium 50 mg orally once daily (QD), and reported stable blood pressure without documented hypotensive episodes. No diabetic complications (e.g., retinopathy and neuropathy) or end-organ damage related to hypertension (e.g., renal impairment) were noted in her medical records. She did not experience nausea, headache, chest distress, diarrhea, or any other symptoms.

On admission, the patient had a body temperature of 38.7°C, a blood pressure of 148/68 mmHg, a pulse of 121 beats per minute, and a respiratory rate of 20 breaths per minute. The patient had large areas of ulceration in the buccal mucosa on both sides of the mouth, especially on the right side, with exposed blood vessels. Initial laboratory blood sample results showed a decrease in the lymphocyte (0.5*10^9^/L), red blood cell (3.14*10^12^/L), and platelet (155*10^9^/L) counts and an increase in the hypersensitive C-reactive protein (8.19 mg/L), serum amyloid A (40.13 mg/L), erythrocyte sedimentation rate (ESA) (60%), procalcitonin (0.359 ng/mL), and interleukin-6 (874.05 pg./mL) levels, indicating an infection (Table 1). In addition, the breast cancer-associated carbohydrate antigen 153 (CA153) index was elevated.

**Table 1:** Laboratory test results of the patient on admission and after treatment.

No.	Variable	Admission	Post-treatment	Normal range
1	White blood cells (*10^9^/L)	6.1	**2.9↓**	3.5–9.5
2	Neutrophil (*10^9^/L)	5.5	1.8	1.8–6.3
3	Lymphocyte (*10^9^/L)	**0.5↓**	**0.8↓**	1.1–3.2
4	Monocyte (*10^9^/L)	0.11	0.23	0.1–0.6
5	Eosinophil (*10^9^/L)	**0↓**	0.02	0.02–0.52
6	Nucleated red cells (*10^12^/L)	0	0	0–0
7	Basophil (*10^9^/L)	0.01	0.02	0.00–0.06
8	Neutrophil ratio (%)	**89.5↑**	62.9	40.0–75.0
9	Lymphocyte ratio (%)	**8.5↓**	27.8	20.0–50.0
10	Monocyte ratio (%)	**1.8↓**	8	3.0–10.0
11	Eosinophilic ratio (%)	**0↓**	0.8	0.4–8.0
12	Nucleated red cells ratio (%)	0	0	0–0
13	Basophil ratio (%)	0.2	0.5	0.0–1.0
14	Red blood cells (*10^12^/L)	**3.14↓**	**2.61↓**	4.30–5.80
15	Hemoglobin (g/L)	115	**97↓**	115–150
16	Hematocrit (%)	**32.8↓**	**27.4↓**	40.0–50.0
17	Average red blood cell volume (fl)	**104.5↑**	**104.8↑**	82.0–100
18	Average hemoglobin content (pg)	**36.7↑**	**37↑**	27.0–34.0
19	Average hemoglobin concentration (g/L)	351	353	316.0–354.0
20	Red blood cell distribution width-CV (%)	11.5	11.7	9.5–19.5
21	Platelet (*10^9^/L)	155	148	125–350
22	Plateletcrit (PCT)	0.12	0.14	0.11–0.28
23	Average platelet volume (fl)	7.7	9.3	5.0–13.0
24	Platelet distribution width-CV (%)	15.8	16.1	12.0–20.0
26	interleukin-6 (IL-6) (pg/mL)	**874.05↑**	9.44	0–10
27	Carbohydrate antigen 153 (CA153)	**32.99↑**	/	0–31.3

“↑” indicate increase compared with normal range, while “↓” denote decrease.

The patient was then transferred to the Department of Respiratory Medicine. Upon admission, the patient underwent a chest CT scan, which revealed no pleural effusion, pneumothorax, or other pleural abnormalities. The results showed normal lung texture and no infection in either lung. Given the patient’s high fever, other infections were considered; therefore, a blood culture was performed immediately, and whole-genome sequencing analysis was performed. Considering the patient’s history of radiotherapy and chemotherapy for breast cancer and high inflammatory markers, the clinician empirically administered piperacillin–tazobactam (4.5 g q8h) in combination with moxifloxacin (0.4 g qd) to provide empirical coverage for ESBL-producing Enterobacterales and synergistic coverage for atypical pathogens. Concurrently, the patient developed sudden high fever, lymphopenia, and immunodeficiency after radiotherapy and chemotherapy for breast cancer, and oseltamivir preventive antiviral treatment was administered. However, the patient’s symptoms persisted, and a dentist was invited for consultation on November 4. The dentist observed that the patient had a large ulcerated area in the buccal and white mucosa. The patient was diagnosed with a severe oral ulcer. Consent was obtained from the patient for the administration of a mouthwash containing tinidazole to dehydrate her mouth to relieve the patient’s mouth ulcer. The clinicians followed the recommendations of the dentist.

**Figure 2:**
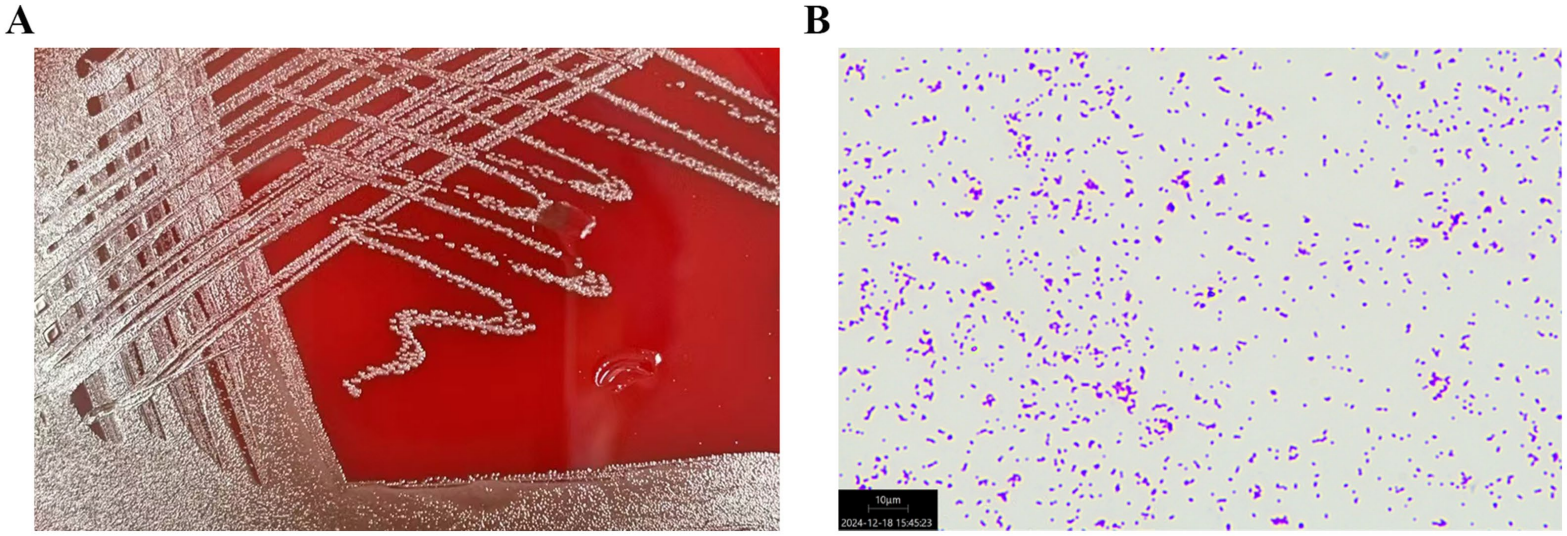
**(A)** *Streptococcus dysgalactiae* ssp. *dysgalactiae* obtained from the patient’s blood sample was cultured on a blood agar plate. **(B)** The microscopic examination of the colonies of *Streptococcus dysgalactiae* ssp. *dysgalactiae* showed purple round spheroids by Gram staining (100×).

Bacterial cultures were performed three times for the blood samples, and the results were positive. Bacteria obtained from the blood samples were cultured on blood agar plates. In addition, the bacterial specimens were first introduced to an atmosphere containing 5% CO_2_ and then incubated for 48 h at 35°C. Microscopic examination revealed the presence of spherical or oval colonies. The colonies were observed in pairs or chains in liquid media. The strain was not motile and did not germinate into spores. The colonies observed on the agar plate demonstrate *α*-hemolysis (Figure 2A).

**Figure 3:**
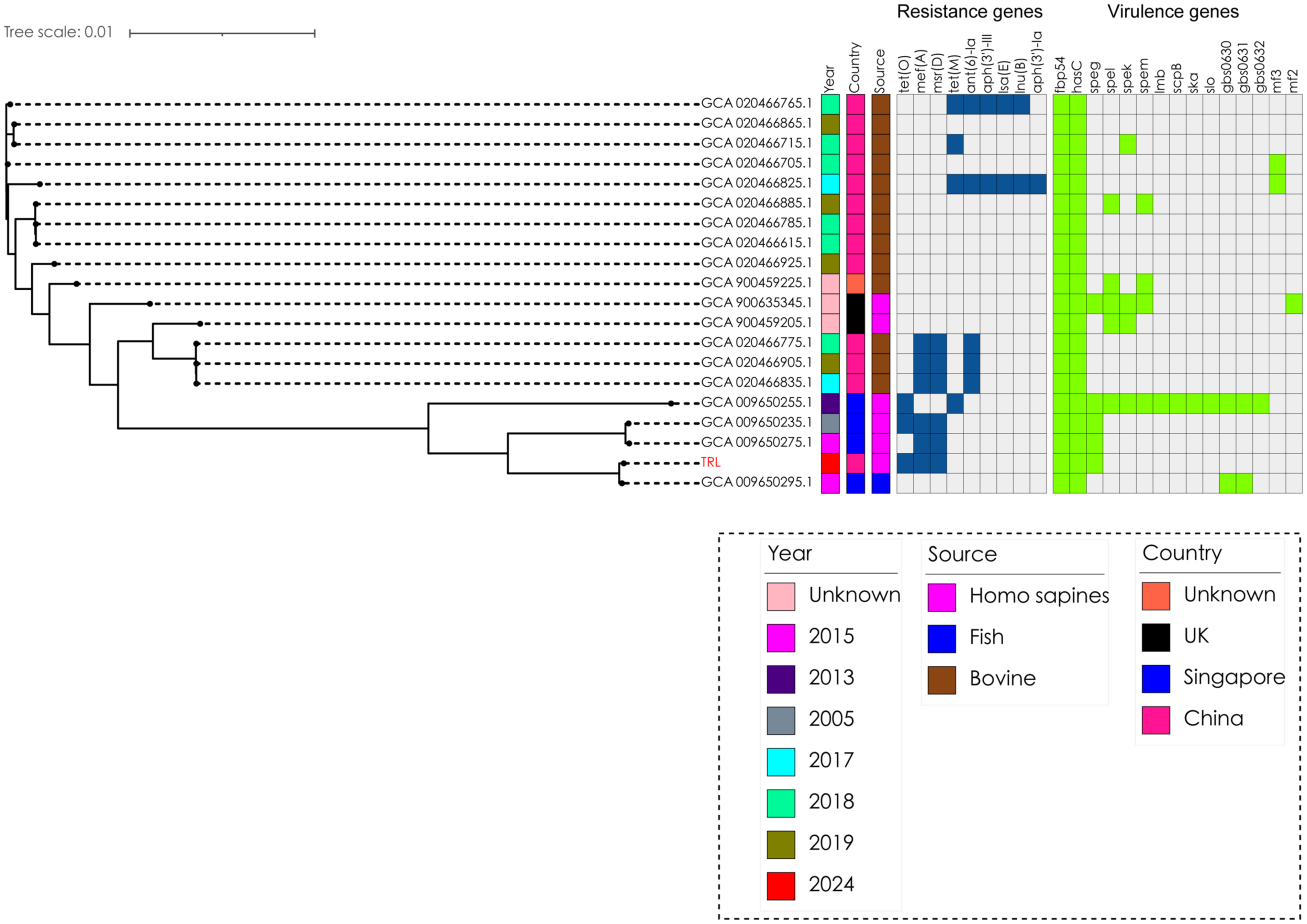
Phylogenetic analysis of 20 *Streptococcus dysgalactiae* ssp. *dysgalactiae* strains. The tree was built with Snippy v4.4.5 and FastTree using RAxML under the GTRGAMMA model with GCA 009650235.1 as the reference strain and visualized with iTOL v5. Isolate names, isolation date, and collection locations are shown for each strain. The TRL strain was used in this study and was labeled as red.

The bacterium was finally identified as SDSD by whole-genome sequencing analysis (specific information is as follows: Gene_number: 2085; Gene_total_length: 1,838,274 bp; Gene_average_length: 881.67 bp; GC_content_in_gene_region: 40.39%; Gene/Genome: 87.13%; Intergenetic_region_length: 271,436 bp; GC_content_in_intergenetic_region: 34.37%; and Intergenetic/Genome: 12.87%). Whole-genome sequencing analysis revealed the presence of a series of resistance genes conferring resistance to different types of antimicrobial agents and virulence factors. The resistance genes included *tet(O)*, *msr(D)*, and *mef(A)* (Figure 3); moreover, the virulence factors included *hasC* for capsule, *fbp54* for fibronectin-binding protein, and *speg* for streptococcal exotoxin G in our strain (Figure 3). Comparative genomic analysis revealed huge diversity in the 20 global SDSD isolates. Only one strain isolated from Singapore exhibited the same resistance profile. In addition, all 20 strains harbored the *hasC* and *fbp54* virulence genes; however, *speg* was identified in only 25% (5/20) of the SDSD isolates. The results showed that the resistance genes included *tet(O)*, *msr(D)*, and *mef(A)*, which are responsible for tetracycline and macrolide resistance (Figure 3). Previous reports have revealed that FBP54 is a cell surface protein that reacts with fibronectin, and *hasC* is involved in the biosynthesis of hyaluronic acid capsules and encodes glucose-1-phosphate uridylyltransferase (14, 15). Based on their function, we speculate that these virulence factor-encoded products may initially adhere to the blood vessel wall, interact with the blood vessel components, and then invade the blood vessel, which further leads to bloodstream infection. Microscopically, the bacterial cells were observed as purple round spheroids by Gram staining, indicating Gram-positive bacteria (100×) (Figure 2B).

On 5 November, the patient was in a state of high fever; however, the ulcerations of the buccal mucosa on both sides of the mouth had improved. Blood culture bacteria showed SDSD, which was considered SDSD bacteremia caused by SDSD. Therefore, antimicrobial therapy with intravenous amoxicillin–clavulanate (2.4 g bid) and moxifloxacin (0.4 g qd) was administered according to whole-genome sequencing analysis and antimicrobial susceptibility results. Antimicrobial susceptibility testing revealed that the bacterial isolates were susceptible to penicillin, ceftriaxone, vancomycin, linezolid, clindamycin, and levofloxacin (Table 2). The whole-genome sequencing analysis results showed that amoxicillin–clavulanate belonged to β-lactam + β-lactamase inhibitors for the treatment of the patient. In this study, the clinician-selected amoxicillin–clavulanate had good treatment outcomes. On 7 November, microbiological examination of the patient’s blood revealed no bacterial or fungal growth. The levels of IL-6, an inflammatory marker, decreased to normal (9.44 pg./mL). Moreover, the patient’s oral ulcers improved, with no other symptoms. The patient showed good prognosis and was discharged on 8 November. On discharge, the patient was instructed to continue oral cefuroxime axetil (0.5 g, bid) for 10 days and visit the hospital every 2 weeks for review. The patient showed a gradual recovery with no relapse of symptoms. In post-discharge interviews, the patient described the infection as ‘more frightening than chemotherapy,’ citing diagnostic uncertainty as a key stressor. She appreciated the care team’s communication clarity and noted adequate emotional support during fever episodes. Satisfaction was assessed through a 5-point Likert scale: 4/5 for clinical competence and 5/5 for psychosocial care. At the 3-month follow-up, the patient had no symptom recurrence. The clinical timeline of the patient is presented in Table 3.

**Table 2:** Antimicrobial susceptibility of *Streptococcus dysgalactiae* ssp*. dysgalactiae* (SDSD) TRL.

Antibiotic	mm/MIC (μg/mL)	Susceptibility
Erythromycin (K-B)	10	R
Tetracycline (K-B)	10	R
Penicillin (MIC)	0.064	S
Ceftriaxone (K-B)	30	S
Vancomycin (K-B)	17	S
Linezolid (K-B)	27	S
Clindamycin (K-B)	22	S
Levofloxacin (K-B)	17	S

S, susceptible; R, resistant.

**Table 3:** Clinical timeline of the patient.

Time point	Clinical event	Diagnostic findings	Intervention/treatment	Outcomes
Day 1	Hospital admission	A large area of ulceration of the buccal mucosa, fever, chills, and fatigue	Physical examination, initial laboratory blood sample, and chest CT, Empirical treatment: antimicrobial therapy with administered intravenous piperacillin–tazobactam (4.5 g q8h) combined with moxifloxacin (0.4 g qd)	Oral ulcer and suspected infection but pneumonia
Day 2	Dental consultation	A large area of ulceration on the buccal mucosa and white mucosa and fever	Added mouthwash of tinidazole	Severe oral ulcer and suspected infection but pneumonia
Day 3	Ongoing treatment	Blood culture: positive for *Streptococcus dysgalactiae* ssp. *dysgalactiae* (SDSD)	Antimicrobial therapy with administered intravenous amoxicillin–clavulanate (2.4 g bid) with moxifloxacin (0.4 g qd)	SDSD bacteremia
Days 4–6	Recovery phase	Fever resolved, oral ulcer resolved, IL-6 normalized, and blood with no bacterial growth	Oral cefuroxime axetil (0.5 g, bid) for 10 days with antibiotics continued	Patient discharged
Three-month follow-up	Outpatient visit	No recurrence of symptoms	Antibiotics completed	Full recovery and no residual symptoms

## Discussion

Breast cancer is one of the most common kind of cancer among women worldwide (16). This cancer originates in breast cells, typically in the ducts or lobules that supply milk (17). Treatment options vary depending on the stage and type of breast cancer and may include surgery, radiation therapy, chemotherapy, hormone therapy, or targeted therapies. Advances in research and treatment have led to improved survival rates and quality of life in many patients (18). Although survival after breast cancer treatment has significantly increased with long-term radiotherapy and chemotherapy (19), aggressive treatments sometimes induce a sharp decline in immune function.

Breast cancer patients, especially those undergoing radiotherapy, chemotherapy, or surgery, often experience immunosuppression, which increases their susceptibility to infection. Patients with immune dysfunction are susceptible to invasive *S. dysgalactiae* infections such as bacteremia, pneumonia, meningitis, keratitis, hepatobiliary tract infections, osteomyelitis, and soft tissue infections (20). However, there have been only a few reports of human infections. One report described an immunocompromised patient with rheumatoid arthritis who developed a prosthetic joint infection caused by SDSD after total knee arthroplasty (21). A 65-year-old man without direct contact with animals developed bloodstream infections and endocarditis caused by SDSD (22). Bloodstream infections are life-threatening conditions in patients with breast cancer, particularly in older individuals, and although the most common risk factor in these patients with tumors is the use of severe neutropenia secondary to myelosuppressive chemotherapy and radiotherapy, other factors may be related to the invasive pathogenicity of microorganisms, as in the case of *S. dysgalactiae* (23). Unfortunately, a college cook died from a bloodstream infection caused by SDSD (24). Furthermore, a previous report indicated that patients with lymphedema and morbid obesity were more predisposed to SDSD bloodstream infections and lower limb cellulitis (25). In addition, SDSD causes upper limb cellulitis in patients in contact with fresh seafood (26). While our study is limited to a single case, it represents the first documented association between SDSD bacteremia and cancer therapy-induced immunosuppression. This finding aligns with emerging evidence, suggesting that non-traditional pathogens may exploit immune vulnerabilities in oncology patients.

The microbiome, comprising a diverse array of beneficial and pathogenic microorganisms, plays a critical role in numerous physiological and pathological processes within the human body, including cancer development (27). Dysbiosis, or microbial imbalance, has also been associated with extraintestinal conditions such as breast disorders through mechanisms involving metabolic interactions and bacterial translocation (28). The female breast provides a nutrient-rich milieu, characterized by adipose tissue, extensive vascular networks, lymphatic vessels, and dispersed lobules and ducts, which support microbial colonization (29). Patients with breast cancer may have an imbalance in intestinal flora after radiotherapy and chemotherapy and are more likely to be infected by external bacteria (30). The gut–breast axis, defined as the migration of gut bacteria via immune cells to secondary lymph nodes, allows these microorganisms to be transported to the breast through the bloodstream or lymphatic system (31). While certain bacteria may occasionally penetrate the intestinal epithelium and rapidly reach the breast tissue, the majority remain confined to the intestinal lumen and mucosa (32). Interestingly, specific bacterial species have a propensity to thrive in the unique tumor microenvironment of breast cancer, where disrupted tumor vasculature and heightened endothelial permeability facilitate the infiltration of pathogenic bacteria (33).

*Streptococcus dysgalactiae* has been classified into two primary subspecies, SDSE and SDSD, both of which are capable of causing central nervous system infections in animals and humans (34). SDSE has been discovered in various animal hosts, including wild animals (cows, horses, sheep, and pigs) and livestock (35). Meanwhile, it has been known as a more significant human pathogenic bacterial microorganism (36). The clinical spectrum of SDSE is similar to that of *S. pyogenes*. Most human infections caused by *S. dysgalactiae* are linked to SDSE, including bacteremia, skin and soft tissue infections, meningitis, endocarditis, and pharyngitis (37–40). A previous case report described a skin incisional wound infection caused by SDSE after breast cancer surgery (41). Another study has reported SDSE-induced streptococcal toxic shock syndrome in a patient with breast cancer (19).

SDSD is predominantly correlated with bovine mastitis and is considered a pathogen that strictly influences animals (42). One study has reported that SDSD induced neonatal mortality in pups (43). In addition, SDSD causes high mortality rates and systemic granulomatous inflammatory diseases in infected fish (44, 45). A previous study evaluated the *in vivo* and *in vitro* potential of SDSD to adhere to the respiratory tract cells in humans (46). It proved that SDSD could be internalized into respiratory tract cells through an initial transport mechanism and demonstrated that SDSD had zoonotic infective abilities in vertebrate hosts, including humans. Although SDSD is principally considered an animal-derived pathogen, the risk of infection with SDSD, in terms of zoonotic potential and virulence, is an urgent concern in humans. Therefore, clinicians should be aware that SDSD can cause cross-infection from animals to humans, especially in immunocompromised individuals and those working in fishing or animal industries. Currently, reports of bloodstream infections with SDSD are scarce in patients with breast cancer after radiotherapy and chemotherapy. SDSD may play a significant role in patients with breast cancer by increasing the risk of infection, potentially influencing the tumor microenvironment, and posing diagnostic and therapeutic challenges (47). Further research is needed to elucidate its impact on breast cancer progression and develop strategies for better management of affected patients.

In the present case, the patient with breast cancer developed a sudden high fever due to SDSD bacteremia, which was triggered by a blood blister puncture by a toothpick in the patient’s mouth. Notably, because patients with a history of radiotherapy and chemotherapy for breast cancer have an immune deficiency, SDSD bacteria may be transmitted to the whole body through the blood when a toothpick punctures blood blisters in the mouth. Therefore, SDSD is an emerging pathogen capable of causing infections in immunocompromised patients with cancer. Physicians must recognize the diagnostic ambiguities and heterogeneous clinical manifestations associated with rare bloodstream infections, especially in resource-constrained settings where delayed identification may exacerbate therapeutic complexities. Furthermore, radiotherapy and chemotherapy for breast cancers often lead to neutropenia and mucosal barrier injury, increasing susceptibility to bacterial and fungal infections (48). Prophylactic antimicrobials can prevent pneumonia in high-risk patients after esophageal cancer surgery (49).

Our early inability to rapidly identify the causative organism was due to the extreme rarity of SDSD in humans. We initially misidentified it as *Streptococcus oralis*. It is worth mentioning that *S. dysgalactiae* has been traditionally separated into *α*-hemolytic SDSD, which is an animal pathogen, and β-hemolytic SDSE, which infects humans. Nevertheless, distinguishing between these two strains of *S. dysgalactiae* may not be simple. In the present case, the bacterial sample was identified only as *S. dysgalactiae* by VITEK-2 and matrix-assisted laser desorption ionization–time-of-flight mass spectrometry, with SDSD and SDSE each accounting for 50%. However, the bacterial subtype could not be determined, and whole-genome sequencing analysis was required to determine whether it was SDSD or SDSE. Whole-genome sequencing analysis later confirmed SDSD as the definitive pathogen, distinguishing it from phenotypically similar streptococci. This case underscores the iterative nature of managing rare infections in immunocompromised hosts. Early broad-spectrum therapy balanced the need for coverage against diagnostic uncertainty, while WGS-enabled precision allowed rapid de-escalation—a paradigm applicable to resource-limited settings.

Studies have shown that antifungals (e.g., fluconazole) can reduce the incidence of fungal infections in patients with neutropenia (50). Although effective, prophylactic antimicrobials must be used judiciously to avoid antibiotic resistance and adverse effects. Radiotherapy and chemotherapy can cause oral mucositis, xerostomia, and dental complications (51). Maintaining rigorous oral hygiene can mitigate these risks and improve the outcomes of patients with cancer (52). Professional dental evaluations and interventions before starting cancer therapy are recommended to address preexisting dental issues. Moreover, understanding the drug resistance and virulence genes of bacteria through whole-genome sequencing analysis can better guide clinicians in antimicrobial therapy. The lack of case reports regarding SDSD bloodstream infections made the treatment approach for the patient difficult in this case; however, prompt treatment was administered with a favorable result.

Despite its novel insights, this study has several methodological constraints. First, this was a retrospective design. Second, while the single-case framework is justified by the extreme rarity of SDSD in older patients with breast cancer, our findings cannot establish causality between chemoradiotherapy and SDSD tropism, nor can they generalize risk factors to broader populations. Finally, although whole-genome sequencing analysis clarified subspecies identity and excluded resistance genes, functional validation of virulence determinants was lacking, limiting mechanistic insights into host–pathogen interactions. To address these limitations, future studies should prioritize prospective cohorts integrating mucosal microbiome metagenomics, experimental models to test zoonotic transmission pathways, and rapid SDSD-specific diagnostics informed by cross-species genomic databases. Such efforts will advance both biological understanding and clinical management of emerging pathogens in immunocompromised hosts.

## Conclusion

In this study, we reported that bloodstream infections caused by SDSD in patients with breast cancer after radiotherapy and chemotherapy are rare and difficult to diagnose early. Microbial culture examination and whole-genome sequencing analysis should be performed on time, and antimicrobial drugs should be administered early to control the disease as soon as possible to avoid death.

## Data Availability

The datasets presented in this study can be found in online repositories. The names of the repository/repositories and accession number(s) can be found: https://www.ncbi.nlm.nih.gov/genbank/, PRJNA1219977.
